# Volume explored by a branching random walk on general graphs

**DOI:** 10.1038/s41598-019-51225-6

**Published:** 2019-10-30

**Authors:** Ignacio Bordeu, Saoirse Amarteifio, Rosalba Garcia-Millan, Benjamin Walter, Nanxin Wei, Gunnar Pruessner

**Affiliations:** 1 Department of Mathematics, Imperial College London, London, SW7 2AZ UK; 2 Centre for Complexity Science, Imperial College London, London, SW7 2AZ UK; 3 Present Address: DAMTP, Centre for Mathematical Sciences, University of Cambridge, Cambridge, CB3 0WA UK; 4 The Wellcome Trust/Cancer Research UK Gurdon Institute, University of Cambridge, Cambridge, CB2 1QN UK

**Keywords:** Complex networks, Statistical physics

## Abstract

Branching processes are used to model diverse social and physical scenarios, from extinction of family names to nuclear fission. However, for a better description of natural phenomena, such as viral epidemics in cellular tissues, animal populations and social networks, a spatial embedding—the branching random walk (BRW)—is required. Despite its wide range of applications, the properties of the volume explored by the BRW so far remained elusive, with exact results limited to one dimension. Here we present analytical results, supported by numerical simulations, on the scaling of the volume explored by a BRW in the critical regime, the onset of epidemics, in general environments. Our results characterise the spreading dynamics on regular lattices and general graphs, such as fractals, random trees and scale-free networks, revealing the direct relation between the graphs’ dimensionality and the rate of propagation of the viral process. Furthermore, we use the BRW to determine the spectral properties of real social and metabolic networks, where we observe that a lack of information of the network structure can lead to differences in the observed behaviour of the spreading process. Our results provide observables of broad interest for the characterisation of real world lattices, tissues, and networks.

## Introduction

Modern models of disease propagation incorporate spatial interaction by allowing a pathogen to be passed on only to the neighbours of an infected host^1,2^. A virus may multiply at a host cell and then infect any of the neighbouring ones at random^3^. The total number of infected cells therefore corresponds to the number of distinct sites visited by a branching random walk (BRW)^4^, also referred to as the Branching Wiener Sausage^5,6^. In this process active random walkers spontaneously produce descendants that carry on hopping from site to site. At the same time, the walkers are subject to spontaneous extinction, for example, by immune-response, healing or decay. The average number of descendants produced during any of these events, branching and extinction, is known to control a transition from a subcritical phase, where the disease ultimately infects only a finite number of sites, to a supercritical phase, where the exponential growth of the virus eventually engulfs almost all available tissue^7^. The expected fraction of distinct sites visited or the size of the epidemic outbreak can be seen as the order parameter of the process.

The characterisation of the distribution of distinct sites visited by a BRW is a long-standing problem of branching processes and random walk theory^4,8,9^. Exact results have been obtained for one-dimensional systems^8^. However, extending such results to higher dimensional lattices and networks is met with major technical obstacles, some of which have been addressed over the past decade^4,10,11^.

In the present work, we characterise analytically and, to confirm our findings, numerically the epidemic spreading in general graphs, including regular lattices, fractal, and artificial and real complex networks, at the onset of epidemics. At this point fluctuations are of crucial importance, dominating the dynamics.

## The Model

We model the epidemic as a Poisson process by considering a reaction-diffusion system of a population of active (mobile, branching, spawning) walkers that hop from their current location **x** on a graph to any adjacent site **y** with rate *H*, and have occupation numbers *n*_**x**_. Walkers are further subject to two concurrent Poisson processes, namely extinction with rate *e* and binary branching with rate *s*, thereby producing descendants, which are indistinguishable from their ancestors.

**Figure 1:**
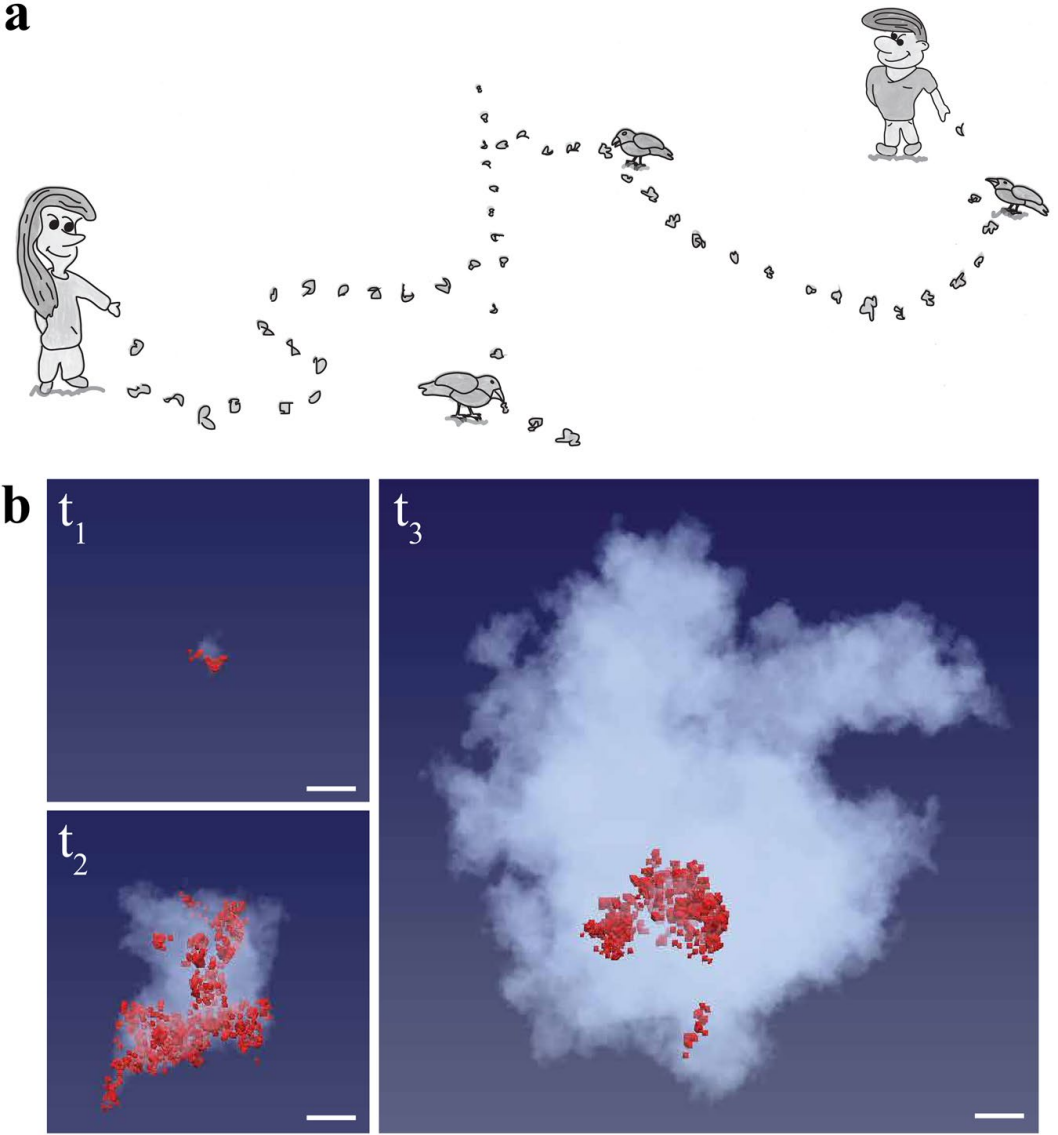
Tracing the path. (**a**) The active walkers, Hänsel and Gretel, leave a trace of breadcrumbs along their way to mark the path they have taken. Birds slowly remove the breadcrumbs, as if they were subject to decay (regularisation, see main text). (**b**) Time evolution of branching random walkers (red) and the cloud of visited sites on a 3d regular lattice at times ${t}_{1} \sim {10}^{2}$, ${t}_{2} \sim {10}^{3}$, and ${t}_{3} \sim {10}^{4}$. Scale bars are equal for all time points.

To extract the number of distinct sites visited, we introduce an immobile tracer particle species with occupation numbers *m*_**x**_. They are spawned as offspring by the active walkers with rate *γ* at the sites they are visiting, thereby leaving a trail of tracers behind, similar to the breadcrumbs left by Hänsel and Gretel^12^, Fig. 1a. We impose the constraint that at most a single tracer can reside at any given site, which means that the spawning of a tracer is suppressed in the presence of another tracer. It is that suppression that generates significant complications from the point of view of the stochastic process. Yet, only with this restriction in place is the number of tracers a measure of the number of distinct sites visited by the walkers, as pictured by the *cloud* of visited sites in Fig. 1b.

There is no interaction between active and tracer particles, other than at the spawning of immobile tracers by active walkers. In principle, the spawning (attempt) rate *γ* has to diverge in order to mark every single site visited by the walkers. However, it turns out that this limit is irrelevant as far as the asymptotic features of this process at large system sizes and long times are concerned^5^.

By definition, the sets {*n*} and {*m*} of occupation numbers *n*_**x**_ and *m*_**x**_, respectively, for each site **x** of a given graph, are Markovian and a master equation can be written for the joint probability ${\mathscr{P}}(\{n\},\{m\};t)$ to find the graph in a certain configuration of occupation numbers at time *t*

1 $$\dot{{\mathscr{P}}}={\dot{{\mathscr{P}}}}_{s}+{\dot{{\mathscr{P}}}}_{e}+{\dot{{\mathscr{P}}}}_{\varepsilon ^{\prime} }+{\dot{{\mathscr{P}}}}_{H}+{\dot{{\mathscr{P}}}}_{\gamma },$$

where $\dot{{\mathscr{P}}}$ corresponds to the time derivative of the (joint) probability ${\mathscr{P}}(\{n\},\{m\};t)$, and the terms on the right-hand side, ${\dot{{\mathscr{P}}}}_{\bullet }={\dot{{\mathscr{P}}}}_{\bullet }(\{n\},\{m\};t)$, indicate the contributions from branching *s*, extinction of active walk*e*rs *e* and tracer particles *ε*′, hopping *H* and deposition *γ*, respectively (see Sec. S1 for details). We constructed a statistical field theory from the master Eq. (1) using the ladder operators introduced by Doi^13^ and Peliti^14^ (methods Sec. V A). To regularise the propagators of the immobile particles in the field theory, we allow for the extinction of immobile particles with rate *ε*′ in Eq. (1), not dissimilar to the birds that foiled Hänsel and Gretel’s plans (Fig. 1a). The propagators for active and tracer particles do not renormalise, and the limit *ε*′ → 0 is taken before any observable is evaluated. Through field-theoretic renormalisation in dimensions *d* = 4 − *ε* we can then determine the exact scaling behaviour of the number of distinct sites visited by the walkers.

The branching process described by Eq. (1) has three regimes, as becomes evident in the field-theoretic formulation, where a *net* extinction rate *r* = *e* − *s* appears. This net extinction rate is not renormalised in the field-theory and therefore no mass shift appears. The BRW is subcritical for *r* > 0, critical for *r* = 0 (onset of epidemics) and supercritical for *r* < 0. Hereafter, we focus on the critical case, where fluctuations dominate the dynamics, and the behaviour becomes unpredictable and highly volatile. Furthermore, for both analytical and numerical computations we consider the initial condition of a single walker at *t* = 0. Extensions to different initial conditions are straight-forward.

## Results for Regular Lattices

Following the field theoretic approach (details in Secs. V A and Sec. S2) of the bulk critical behaviour in the continuum limit, where hopping is replaced by diffusion by introducing a diffusion constant *D*, we find that in the thermodynamic limit at long times *t*, the expected number of distinct sites visited or the volume explored, 〈*a*〉(*t*, *L*), scales like *t*^(*d*−2)/2^ in dimensions *d* < 4. In dimensions *d* < 2 this volume remains finite in large *t*. The scaling of the *p*-th moment of the number of distinct sites visited follows,

2a $$\langle {a}^{p}\rangle (t,L)\propto {t}^{(pd-\mathrm{2)/2}}\,{\rm{for}}\,Dt\ll {L}^{{\rm{2}}}$$

2b $$\langle {a}^{p}\rangle (t,L)\propto {L}^{(pd-\mathrm{2)}}\,{\rm{for}}\,Dt\gg {L}^{2}$$

in *d* < 4 provided that *pd* − 2 > 0. The gap-exponent^15^ of 〈*a*^*p*+1^〉/〈*a*^*p*^〉 for the sc*a*ling in *L*, which can be thought of as the effective dimension of the cluster of visited sites, is therefore *d* in dimensions less than *d*_*c*_ = 4.

**Figure 2:**
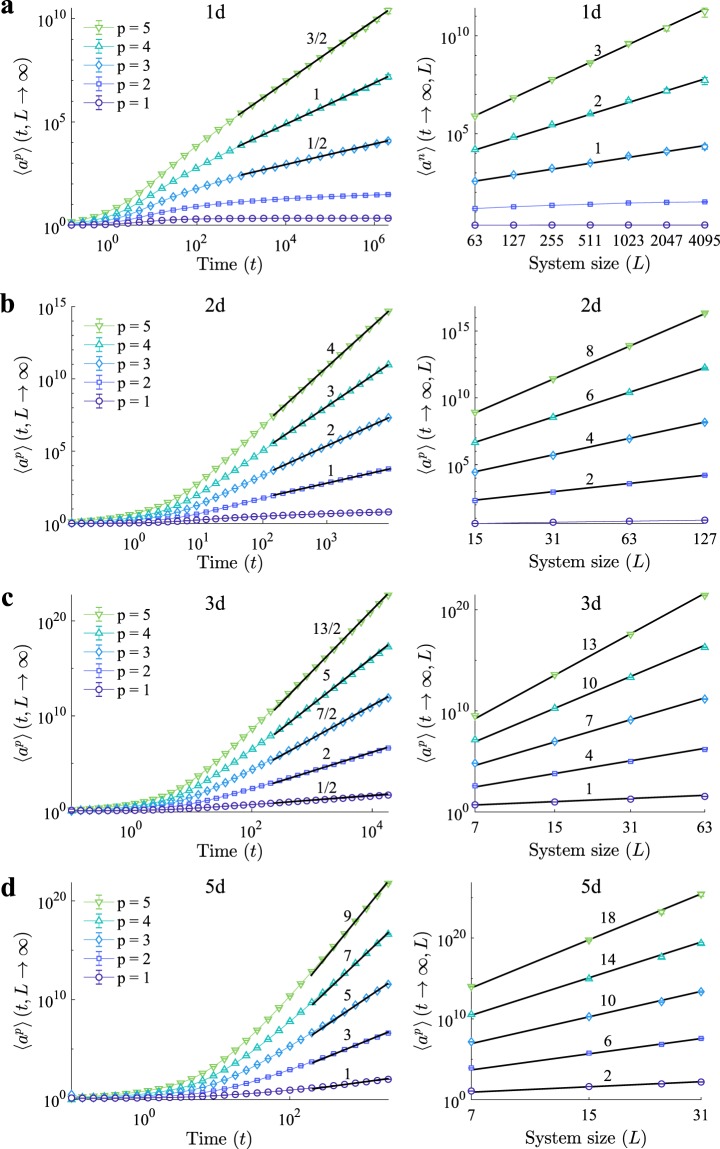
Distinct sites visited on regular lattices. Scaling of the moments of the numbers of distinct sites visited in time (left) and system size (right) for (**a**), 1d, (**b**), 2d, (**c**), 3d, and (**d**) 5d regular lattices. Solid black lines represent the theoretical exponents given by Eq. (2) for *d* < 4, and Eq. (3) for *d* > 4. Simulations parameters: *H* = 0.1, *s* = *e* = 0.45, *ε*′ = 0, and *γ* → ∞.

These results describe the numerical observations on regular lattices in dimensions *d* = 1, 2 and 3 (see Sec. V B 1), as shown in Fig. 2a–c, respectively, where, after an initial transient, the moments scale according to Eq. (2) in time and system size (see Tables S1 and S2). The process is *free* beyond *d*_*c*_ = 4 dimensions, where the probability of any walker or any of its ancestors or descendants ever to return to a previously visited site drops below unity, and the scaling becomes independent of the dimension,

3a $$\langle {a}^{p}\rangle (t,L)\propto {t}^{2p-1}\,{\rm{for}}\,Dt\ll {L}^{{\rm{2}}}$$

3b $$\langle {a}^{p}\rangle (t,L)\propto {L}^{4p-2}\,{\rm{for}}\,Dt\gg {L}^{{\rm{2}}}$$

with logarithmic corrections in *d* = *d*_*c*_ = 4. The gap-exponent in dimensions greater than *d*_*c*_ = 4 is thus 4, as confirmed by numerical observations in dimension *d* = 5 (see Fig. 2d and Table S2). As correlations become irrelevant, this is usually referred to as mean-field behaviour. The set of sites visited may thus be regarded as a four-dimensional object, projected into the *d*-dimensional lattice considered. Focusing on dimensions below *d*_*c*_ = 4, the distribution of the number of distinct sites visited, *a*, follows a power law,

4 $${\mathscr{P}}(a)=A{a}^{-\mathrm{(1}+\mathrm{2/}d)}{\mathscr{G}}(a/{a}_{c})$$

with metric factor *A* and cutoff *a*_*c*_~(*Dt*)^*d*/2^ for *Dt* ≪ *L*^2^ and *a*_*c*_ ~ *L*^*d*^ otherwise. These results show how increasing the dimensionality of the lattice promotes the appearance of larger events, evidencing the relevance of dimension on the spreading.

**Figure 3:**
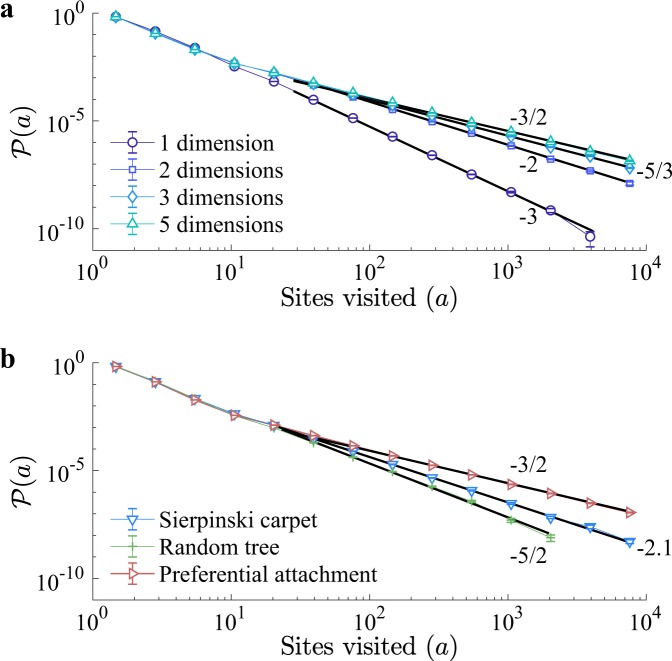
Probability distribution of the number of visited sites, (**a**) for regular lattices of dimensions *d* = 1, 2, 3 and 5, and for (**b**) Sierpinski carpet, random tree, and a preferential attachment (scale-free) networks. The solid black lines represent the predicted scaling given by Eq. (4). Simulations parameters: *H* = 0.1, *s* = *e* = 0.45, *ε*′ = 0, and *γ* → ∞.

In dimensions *d* ≥ *d*_*c*_ = 4 the resulting scaling of the distribution is that of Eq. (4) at *d* = 4, where the probability distribution decays like *a*^−3/2^. Numerically, we recorded, for each realisation, the total number of distinct sites visited by the process in order to construct the distribution, ${\mathscr{P}}(a)$, of sites visited. The numerical results coincide with our theoretical predictions, as shown in Fig. 3a.

The exponents found above for *d* = 1 are in agreement with the exact solution by Ramola *et al*.^8^, where ${\mathscr{P}}(a)$ decays as *a*^−3^. In two dimensions the power-law tail decays as *a*^−2^, which coincides with the decay of the 2d convex hull area distribution^4^.

## Extension to General Graphs

In the field theoretic approach followed to find the scaling in Sec. II the spatial dimension of the lattice enters only in as far as its spectral dimension is concerned, which characterises the density of eigenvalues of the Laplace operator on the graph given. Our results extend naturally to all translational invariant lattices and graphs, by replacing the dimension *d* of the lattice in Eqs (2)–(4) by the spectral dimension *d*_*s*_ of the graph, as detailed in Sec. S5. This holds true more generally as long as the lattice Laplacian itself does not undergo renormalisation, *i.e*. in the absence of an anomalous dimension^16^. In the study of networks the number of nodes *N*, is a more natural measure of the size of the network than the linear size *L*. Using *L* ~ *N*^1/*d*^_*s*_ we can write the scaling of the BRW in time and number of nodes as

5a $$\langle {a}^{p}\rangle (t,N)\propto {t}^{(p{d}_{s}-\mathrm{2)/2}}\,{\rm{for}}\,Dt\ll {N}^{2/{d}_{s}}$$

5b $$\langle {a}^{p}\rangle (t,N)\propto {N}^{(p-\mathrm{2/}{d}_{s})}\,{\rm{for}}\,Dt\gg {N}^{2/{d}_{s}}.$$

**Figure 4:**
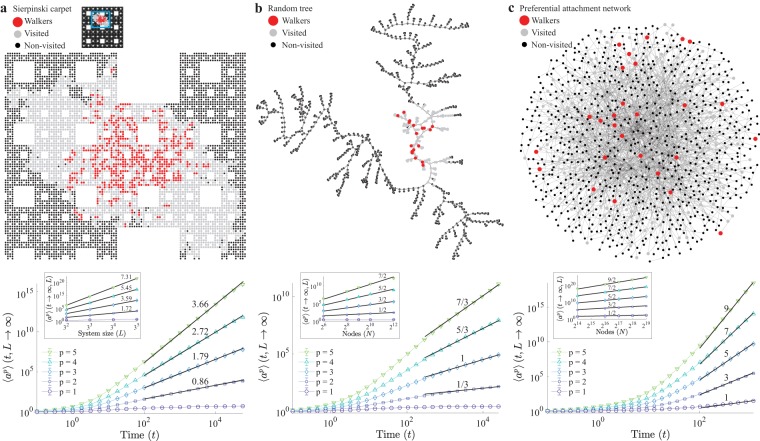
Scaling on general graphs on (**a**), the Sierpinski carpet, (**b**), random tree and, (**c**), preferential attachment networks. The top row shows representative states (full Sierpinski carpet shown on inset), indicating walkers (red), visited sites (grey) and non-visited sites (black). The bottom row shows the scaling of moments of the number of distinct sites visited as a function of time, and linear system size (inset), or number of nodes, in the case of networks. The solid black lines represent the predicted scaling from Eq. (5). Simulation parameters: *H* = 0.1, *s* = *e* = 0.45, *ε*′ = 0, and *γ* → ∞.

Here, the gap-exponent for the scaling in number of nodes is always unity. This extension to graphs allowed us to predict the behavior of the BRW spreading in both artificial networks relevant for social and biological sciences, and complex systems in general^2,17–19^, as well as real networks. To illustrate this, we considered first the Sierpinski carpet (SCs) (Fig. 4a, methods Sec. V B 2), and random trees (RTs) (Fig. 4b and methods Sec. V B 3). Both of these graphs are widely applied in the context of porous media^20^ and percolation^21^, and have known spectral dimension: *d*_*s*_ ≈ 1.86 for the SC^22^, and *d*_*s*_ = 4/3 for RTs^23^. Considering Eq. (2) with $d={d}_{s}$, for the SC, and (5) for the RT we obtain accurate predictions for the spreading dynamics as confirmed by numerical simulations, Fig. 4d,e. These theoretical predictions extend also to the distribution of visited sites (see Fig. 3b), by setting *d* = *d*_*s*_ in (4).

Furthermore, we studied the BRW behaviour on a class of scale free networks^24^. Since their introduction, scale free graphs have been observed to describe a plethora of natural phenomena, including the World-Wide-Web^25^, transportation^26^, and metabolic networks^27^, to name but a few. We considered a preferential attachment scheme^24^ (Fig. 4c, see methods Sec. V B 4), to construct networks with power-law degree distribution (Fig. S1). The existence of a finite spectral gap in these networks, which indicates slow decay of return times^28,29^, suggested that the BRW process is bound to exhibit mean-field behaviour, i.e. *d*_*s*_ ≥ 4. This was confirmed by numerical simulations, where the probability distribution of visited sites (Fig. 3b) has a power-law decay with exponent −1.52(2) ≈ −3/2, and the scaling in time and system size (Fig. 4f and Table S1) follow mean-field behaviour as predicted by (3) for *d*_*s*_ = 4.

**Figure 5:**
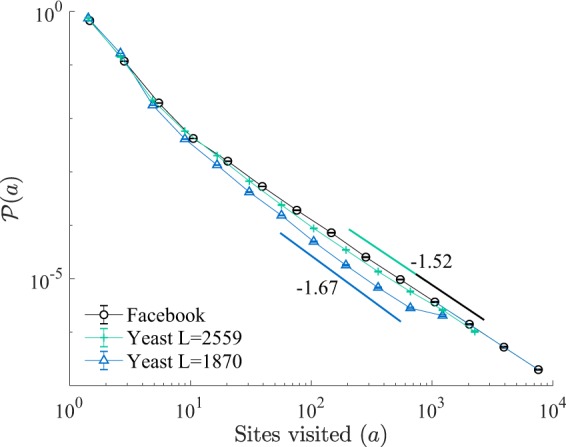
Probability distribution of number of distinct sites visited ${\mathscr{P}}(a)$, for the Facebook network (*L* = 63730 nodes)^30^, and yeast protein interaction networks with *L* = 1870^31^, and *L* = 2559 nodes^32^. The data was obtained from simulations of the BRW on each graph, with parameters *H* = 0.1, *s* = *e* = 0.45, *ε*′ = 0, and *γ* → ∞.

The spectral dimension gives information on the behaviour of dynamical processes on graphs. Here we use the BRW to characterise real-world networks through the power-law decay of the distribution of visited sites ${\mathscr{P}}(a)$, which according to Eq. (4) is ${a}^{-\mathrm{(1}+\mathrm{2/}{d}_{s})}$ provided *d*_*s*_ ≤ 4. For example, the BRW exhibits near mean field-behaviour on a subset of the Facebook network, which has been characterised as scale-free^30^. Hence, we derived a large effective (spectral) dimension, *d*_*s*_ = 3.9(1), indicating a fast spreading of the viral process in this network (see Fig. 5).

We should emphasize that the spectral dimension is sensitive to changes in network topology and connectivity. To exemplify this we have considered two publicly available datasets for the yeast protein interaction network (see Fig. 5). We found that even though both network describe subsets of the same biochemical network, namely the complete yeast protein interactome, the spectral dimensions in both cases are significantly different, *d*_*s*_ = 3.0(1) for the network with *N* = 1870 nodes^31^, and *d*_*s*_ = 3.8(1) for the larger network of *N* = 2559 nodes^32^, leading to differences in properties of the spreading process among the two. The discrepancy points to differences in the connectivities of both networks and shows the importance of having access to the complete network in order provide a reliable analysis of its properties, which may have biological implications^33,34^.

## Outlook

The results presented above for the binary branching process, where walkers branch into exactly two new walkers, apply equally to more general branching processes, where the number of offspring in each birth event is given by a distribution (for details see Sec. S4). This can be seen, for example, in real-world scenarios where a single infected individual or device infects a whole neighbourhood around them, or in the case of signal propagation in protein networks, where the activation of one node (or chemical reaction) can in turn activate a whole fraction of its neighboring nodes.

While the scaling behaviour does not depend on the initial position **x**_0_ of a walker, provided it is located in the bulk and remains there as the thermodynamic limit is taken, the field theory has to be adjusted to account for more complicated boundary conditions^5^ or the walker starting close to any such boundary. It may also be interesting to consider the case of initialising each site with an independent Poisson distributions of walkers^35^.

The approach followed in the present work provides a quantitative measure to explore and determine the spectral dimension of artificial and real networks. This is of particular interest when the spectral dimension is greater or equal to 2, where the traditional approach of exploring graphs, based on simple random walks^29,36^, fails. When simulating the BRW we made the observation that robust scaling is more easily obtained on small lattices if the hopping rate *H* is clearly smaller than the rates of branching *s* and extinction *e*. For large values of the hopping rate particles leave the system during the initial transient, as seen in Fig. 2, thus boundary effects appear before any robust scaling can be observed. In graphs such as the PA network (Fig. 4), that does not have any boundaries, these artefacts are much less pronounced. In summary our results shed new light on the properties of spatial branching processes on general graphs, and their applicability in the study of real complex networks, and provide observables of broad interest for the characterisation of real world lattices, tissues, and networks.

## Methods

### Field theory of the BRW

In order to derive the main results for the scaling of distinct sites visited by the BRW (Sec. II) we work along established lines^37^, casting the master equation in a field theory of the annihilation fields *ϕ*(**x**, *t*) and $\psi ({\bf{x}},t)$ for the active and the immobile particles, respectively, and of the corresponding (Doi-shifted) creation fields $\tilde{\varphi }({\bf{x}},t)$ and $\tilde{\psi }({\bf{x}},t)$. The governing Liouvillian ${\mathcal L} ={ {\mathcal L} }_{0}+{ {\mathcal L} }_{1}$ consists of a harmonic part,

6 $${ {\mathcal L} }_{0}(\varphi ,\psi ,\tilde{\varphi },\tilde{\psi })=-\tilde{\varphi }{\partial }_{t}\varphi +D\tilde{\varphi }{\nabla }^{2}\varphi -r\tilde{\varphi }\varphi -\tilde{\psi }{\partial }_{t}\psi -\varepsilon ^{\prime} \tilde{\psi }\psi +\tau \tilde{\psi }\varphi ,$$

and a non-linear part,

7 $${ {\mathcal L} }_{1}(\varphi ,\psi ,\tilde{\varphi },\tilde{\psi })=s{\tilde{\varphi }}^{2}\varphi +\sigma \tilde{\psi }\tilde{\varphi }\varphi -\lambda \tilde{\psi }\psi \varphi -\xi {\tilde{\psi }}^{2}\psi \tilde{\varphi }\varphi -\kappa \tilde{\psi }\psi \tilde{\varphi }\varphi -\chi {\tilde{\psi }}^{2}\psi \varphi ,$$

where we have taken the continuum limit. The space and time integrated Liouvillian produces the field-theoretic action ${\mathscr{A}}=\int \,{{\rm{d}}}^{d}x{\rm{d}}t\, {\mathcal L}$, whose exponential ${{\rm{e}}}^{{\mathscr{A}}}$ enters into the path integral formulation. The couplings in the Liouvillian are related to the rates in the master equation as follows: *D* is a diffusion constant *D* = *H*Δ*x*^2^, where Δ*x* is the lattice spacing, and $H\propto \Delta {x}^{-2}$ when the limit Δ*x* → 0 is taken, in order to maintain finite diffusivity. At bare level the non-linear couplings, with the exception of the branching rate *s*, are equal to spawning rate *γ*, i.e. *τ* = *σ* = *λ* = *ξ* = *κ* = *χ* = *γ*. This follows from translating the master Eq. (1) into field-theoretic language (see Sec. S1 for details).

At the same time the *net* extinction rate *r* = *e* − *s*, the field-theoretic mass of the walkers, has to be kept finite. In this parameterisation, there are three regimes, as described in the main text: a subcritical one for *r* > 0, a critical for *r* = 0 and a supercritical for *r* < 0. In the field theory, all large scale (infrared) phenomena will be controlled by *r* → 0^+^, which corresponds to the onset of epidemics, the limit studied in this work. The mass of the tracers, *ε*′, serves merely as a regularisation, and is removed by taking the limit *ε*′ → 0. The bare transmutation rate *τ*, corresponding to $\gamma$ on the lattice, and the bare branching rate *s* of the active particles (*s* on the lattice) are the two processes that we expect will govern all infrared behaviour in all dimensions and are therefore assumed to be dimensionless. These two choices determine the engineering dimension^38^ of all other bare couplings, resulting in *ξ*, *κ* and *χ* being infrared irrelevant. Together with *λ*, these four couplings are due to the suppression of the spawning of tracers when a site is occupied already. At the upper critical dimension, *d*_*c*_ = 4, the coupling *λ* is marginally relevant, being infrared irrelevant above and relevant below. The minimal subtraction scheme^38^ we have used will produce results in terms of *ε* = 4 − *d*.

The Liouvillian constructed above is the object that allows the exact calculation of the scaling exponents of the *p*-th moment of the volume explored by a branching random walk 〈*a*^*p*^〉(*t*, *L*), in time *t*, and linear system size *L*. Initialising the system at time *t*_0_ = 0 with a single active walker at position **x**_0_, field-theoretically implemented by the creation field $\tilde{\varphi }({{\bf{x}}}_{0},\mathrm{0)}$, the ensemble average 〈*a*〉(*t*, *L*) of the volume explored by the BRW is determined by

8 $$\langle a\rangle (t,L)=\int {{\rm{d}}}^{d}x\,\langle \psi ({\bf{x}},t)\tilde{\varphi }({{\bf{x}}}_{0}\mathrm{,0)}\rangle ,$$

where the density of tracers particles at position **x** and time *t* > 0 is measured by *ψ*(**x**, *t*) and integrated over all space. Similarly^5^, higher moments are dominated by integrals of the form

9 $$\langle {a}^{p}\rangle (t,L)\sim \int \,{{\rm{d}}}^{d}{x}_{p}\ldots {{\rm{d}}}^{d}{x}_{1}\,\langle \psi ({{\bf{x}}}_{p},t)\ldots \psi ({{\bf{x}}}_{1},t)\mathop{\varphi }\limits^{ \sim }({{\bf{x}}}_{0},0)\rangle ,$$

or equivalently, by evaluating the Fourier transform at spatial momentum **k** = 0. These are functions of the couplings introduced above, but to leading order not of the walker’s initial position **x**_0_, provided it is located in the bulk. We implement this numerically by always placing the walker initially at the centre site of odd-sized regular lattices, see Sec. V B 1. The average 〈•〉 introduced on the right hand side of Eq. (8) correspond to the path integral

10 $$\langle \psi ({{\bf{x}}}_{p},t)\ldots \psi ({{\bf{x}}}_{1},t)\tilde{\varphi }({{\bf{x}}}_{0}\mathrm{,0)}\rangle =\int \,{\mathscr{D}}\Pi \,(\psi ({{\bf{x}}}_{p},t)\ldots \psi ({{\bf{x}}}_{1},t)\tilde{\varphi }({{\bf{x}}}_{0}\mathrm{,0))}{{\rm{e}}}^{\int {{\rm{d}}}^{d}x{\rm{d}}t {\mathcal L} },$$

which measures the *p*-point correlation function of tracers at (**x**_*i*_, *t*), *i* = 1, 2, …, *p* in response to the creation of a walker at (**x**_0_, *t* = 0). Here, the integration measure is ${\mathscr{D}}\Pi ={\mathscr{D}}\varphi \,{\mathscr{D}}\tilde{\varphi }\,{\mathscr{D}}\psi \,{\mathscr{D}}\tilde{\psi }$. Field theoretic renormalisation in dimensions *d* = 4 *−* *ε* then allows us to derive the scaling of the number of distinct sites visited (see Sec. S2 for more details).

### Numerical implementation

In the numerical implementation, an active particle is allowed to diffuse by hopping from the site it resides on to a nearest neighbouring site with rate *H*, branch with rate *s* by placing an identical offspring at the present site or become extinct with rate *e*. Each distinct site visited is recorded, equivalent to taking the limit *γ* → ∞ in the theory. The instantaneous number *a*(*t*, *L*) of distinct sites visited up to time *t* is therefore the number of sites recorded. Parameters were chosen such that *H* + *s* + *e* = 1, *H* was set to 0.1, and *e* = *s* = 0.45. If *M* walkers are present in the system at a given time the waiting time for the next event (hopping, branching or extinction) is determined by −*ln*(1 − *u*)/*M* where $u\in (0,1]$ is a uniformly distributed random variable. For every lattice size we performed 10^6^–10^9^ realisations of the process.

#### Regular, integer-dimensional lattices

The regular lattices studied here are hypercubic *d*-dimensional lattices, characterised by their linear size *L* = 2^*m*^ − 1, *m* ≥ 4, which is chosen to be odd so that it contains a well-defined centre site, on which the single active walker is initially placed. To study finite-size scaling, absorbing boundary conditions were applied. However, we observed that the boundary conditions have no effect on the scaling (data not shown). The numerical results were fitted to a power-law as described in Sec. S6, to obtain the values in Tables S1 and S2.

#### Sierpinski carpet

The Sierpinski carpets were constructed from two dimensional lattices of linear dimension 3^*m*^, *m* ≥ 2. The lattice was divided into 3^2^ equal sub-squares each of size 3^*m*−1^, the central square was removed, leaving 3^2^ − 1 sub-squares. The procedure is iterated over the remaining sub-squares. The spectral dimension of the Sierpinski carpet has been estimated to be *d*_*s*_ = 1.86^22,39^. A random point around the central hole of the fractal was used as the initial location of the walker in every realisation.

#### Random trees

The critical random tree networks^40^ were constructed as a critical Galton-Watson process, where every node has either 0, 1, or 2 descendants, such that the mean degree of the network is 2. We generated networks with 2^6^–2^12^ nodes. These graphs have no closed loops. The spectral dimension of the random tree ensemble is *d*_*s*_ = 4/3^23^. For every realisation of the process, a new random tree was generated, and a node was selected at random as the starting location of the initial walker.

#### Preferential-attachment network

A preferential attachment (PA) network is a class of scale-free networks, characterised by a power-law degree distribution. We followed the Barabási-Albert model of preferential attachment^24^ initialised with a single node to generate networks with 2^12^–2^19^ nodes. The networks have power-law degree distribution with exponent −2.9 and mean degree $\langle k\rangle \,=\,6.3$ (see Fig. S1). For every realisation of the process, a new network was constructed, and a node was selected at random as the starting location of the initial walker.

## Supplementary information

Supplementary material

## Data Availability

The numerical data is available upon request, by contacting I.B., ibordeu@imperial.ac.uk.
