# Supplementary material for: Volume explored by a branching random walk on general graphs
Source: Sci Rep. 2019 Oct 30;9:15590. doi: 10.1038/s41598-019-51225-6 (PMC6821755; doi:10.1038/s41598-019-51225-6)
Supplement: Supplementary file 1 — Supplementary material [file 41598_2019_51225_MOESM1_ESM.pdf]

Supplementary material

Volume explored by a branching random walk on general graphs

Ignacio Bordeu[1,2,*], Saoirse Amarteifio[1,2], Rosalba Garcia-Millan[1,2], Benjamin Walter[1,2], Nanxin Wei[1,2], and Gunnar Pruessner[1,2,†]

[1]Department of Mathematics, Imperial College London, London SW7 2AZ, UK
[2]Centre for Complexity Science, Imperial College London, London SW7 2AZ, UK
[*]ib443@cam.ac.uk
[†]g.pruessner@imperial.ac.uk

S1. MASTER EQUATION FOR THE BRANCHING RANDOM WALK

In the following we describe the contributions to the master Eq. (1) from each of the processes the branching random walk comprises. The contribution to the master equation for the joint probability $\mathcal{P}(\{n\},\{m\};t)$ from the spawning of immobile tracer particles by active walkers must take into account the finite carrying capacity $\bar{m}_0$ of each lattice site. To account for a finite carrying capacity an effective deposition rate is introduced that decays linearly with the number of tracer particles already present at the site of interest [S1],

$$\gamma_{\text{eff}} = \gamma \frac{\bar{m}_0 - m_{\mathbf{x}}}{\bar{m}_0}.$$

To study the number of distinct sites visited $\bar{m}_0$ is set to 1. With this constraint in place, each site visited is *marked* with a tracer particle at most once, so that their total number is that of *distinct* sites visited by the BRW. With these considerations, the contributions to the master equation from deposition of tracer particles read

$$\dot{\mathcal{P}}_\gamma(\{n\},\{m\};t) = \gamma \sum_{\mathbf{x}} \Big((1-(m_{\mathbf{x}}-1))n_{\mathbf{x}}\mathcal{P}(\{n\},\{\dots,m_{\mathbf{x}}-1,\dots\};t) - (1-m_{\mathbf{x}})n_{\mathbf{x}}\mathcal{P}(\{n\},\{m\};t)\Big), \tag{S1}$$

where $n_{\mathbf{x}}$ and $m_{\mathbf{x}}$ correspond to the number of active and immobile particles at site $\mathbf{x}$. The sum $\sum_{\mathbf{x}}$ runs over all lattice sites. The contribution from branching of active walkers reads

$$\dot{\mathcal{P}}_s(\{n\},\{m\};t) = s \sum_{\mathbf{x}} \Big((n_{\mathbf{x}}-1)\mathcal{P}(\{\dots,n_{\mathbf{x}}-1,\dots\},\{m\};t) - n_{\mathbf{x}}\mathcal{P}(\{n\},\{m\};t)\Big). \tag{S2}$$

The contributions from extinction are

$$\dot{\mathcal{P}}_e(\{n\},\{m\};t) = e \sum_{\mathbf{x}} \Big((n_{\mathbf{x}}+1)\mathcal{P}(\{\dots,n_{\mathbf{x}}+1,\dots\},\{m\};t) - n_{\mathbf{x}}\mathcal{P}(\{n\},\{m\};t)\Big) \tag{S3}$$

for active particles, and

$$\dot{\mathcal{P}}_{\epsilon'}(\{n\},\{m\};t) = \epsilon' \sum_{\mathbf{x}} \Big((m_{\mathbf{x}}+1)\mathcal{P}(\{n\},\{\dots,m_{\mathbf{x}}+1,\dots\};t) - m_{\mathbf{x}}\mathcal{P}(\{n\},\{m\};t)\Big) \tag{S4}$$

for immobile particles. Finally, the contribution to the joint probability from the hopping of active walkers reads

$$\dot{\mathcal{P}}_H(\{n\},\{m\};t) = \frac{H}{q} \sum_{\mathbf{x}} \sum_{\mathbf{y}.nn.\mathbf{x}} \Big((n_{\mathbf{y}}+1)\mathcal{P}(\{\dots,n_{\mathbf{x}}-1,\dots,n_{\mathbf{y}}+1,\dots\},\{m\};t) - n_{\mathbf{x}}\mathcal{P}(\{n\},\{m\};t)\Big), \tag{S5}$$

where the sum $\sum_{\mathbf{y}.nn.\mathbf{x}}$ runs over all q nearest neighbouring (nn) sites $\mathbf{y}$ of $\mathbf{x}$.

Combining the contributions from all the subprocesses, the master equation for the joint probability $\mathcal{P} = \mathcal{P}(\{n\},\{m\};t)$ reads

$$\dot{\mathcal{P}} = \dot{\mathcal{P}}_s + \dot{\mathcal{P}}_e + \dot{\mathcal{P}}_{\epsilon'} + \dot{\mathcal{P}}_H + \dot{\mathcal{P}}_\gamma. \tag{S6}$$

as shown in Eq. (1).

S2. FIELD-THEORY OF THE BRW

In the following, we show the details of the field-theoretical calculations performed to obtain the main results of the article, Eqs. (2), (4), and (3). In Sec. S2 A we describe the dimensional analysis of the bare couplings. In Sec. S2 C, we introduce a diagrammatic representation of the propagators and couplings, and in Sec. S2 D we determine the relevant interactions. In Sec. S2 E, we perform the renormalisation of the couplings, and finally calculate the higher order correlations that give rise to the scaling of the moments of the number of distinct sites visited in Sec. S2 G.

A. Dimensional analysis of the bare couplings

To compute the critical dimension of the process described by the Liouvillian $\mathcal{L} = \mathcal{L}_0 + \mathcal{L}_1$, Eqs. (6) and (7), and to extract the relevant interactions, i.e the couplings that remain relevant in every spatial dimension, we study the engineering dimensions (here, represented by $[\cdot]$) of every coupling in the action. We expect that the long range physics in time and space is governed by three processes: diffusion with constant D, branching with rate s, and transmutation with rate τ. Introducing three independent dimensions, namely $\mathtt{A}$, $\mathtt{B}$ and $\mathtt{C}$, we impose

$$[\tau] = \mathtt{A}, \quad [s] = \mathtt{B}, \quad \text{and} \quad [D] = \mathtt{C}. \tag{S7}$$

With $[\mathbf{x}] = \mathtt{L}$, $[t] = \mathtt{T}$, and $[\partial_t] = [D\nabla^2]$ it follows that $\mathtt{T} = \mathtt{CL}^2$ is not an independent dimension. As the action, $\mathcal{A} = \int \mathrm{d}^d x \mathrm{d}t\, \mathcal{L}$, itself must be dimensionless, i.e. $[\mathcal{A}] = 1$, we obtain $[r] = \mathtt{T}^{-1} = \mathtt{CL}^{-2}$ and

$$\left[\tilde{\phi}\right] = \mathtt{B}^{-1}\mathtt{CL}^{-2}, \qquad [\phi] = \mathtt{BC}^{-1}\mathtt{L}^{2-d}, \qquad \left[\tilde{\psi}\right] = \mathtt{A}^{-1}\mathtt{B}^{-1}\mathtt{C}^{2}\mathtt{T}^{-2}, \qquad [\psi] = \mathtt{ABC}^{-2}\mathtt{L}^{4-d} \tag{S8}$$

for the fields in real time and space, such that $[\tilde{\phi}\phi] = [\tilde{\psi}\psi] = \mathtt{L}^{-d}$. The engineering dimensions of the couplings follow:

$$[\lambda] = \mathtt{B}^{-1}\mathtt{C}^2\mathtt{L}^{d-4} \qquad [\sigma] = \mathtt{ABC}^{-1}\mathtt{L}^2 \qquad [\chi] = \mathtt{AL}^d \tag{S9a}$$

$$[\kappa] = \mathtt{CL}^{d-2} \qquad [\xi] = \mathtt{ABC}^{-1}\mathtt{L}^{d+2}. \tag{S9b}$$

Setting $\mathtt{A} = \mathtt{B} = \mathtt{C} = 1$, we find a critical dimension $d_c = 4$, above which all of these interactions become irrelevant. At the critical dimension $d = d_c = 4$ the couplings σ, χ, κ, and ξ remain irrelevant, while λ becomes marginal. To regularise the ultraviolet we work in dimensions $d = 4 - \epsilon < 4$.

As a point of discussion, we note that other choices of independent dimensions are possible, limited only by the symmetries to be preserved. Initially we considered σ, rather than τ to have an independent dimension. The resulting (very messy) field theory depends on the non-universal, bare value of s and produces no renormalisation of τ, which, however, *must* renormalise as $\langle a \rangle (t, L) \sim \tau_{\text{eff}} L^2$ (see Section S2 E) and cannot scale faster than the volume of the system, L^d.

A coupling with independent dimension is saved from changing relevance and thus from possible irrelevance in the infrared limit of large space and long time. The choice of dimensions is therefore a choice of interactions that ultimately govern the infrared. If the stochastic process under consideration takes place on the lattice, this may be determined by taking the continuum limit, provided the process does not possess any competing scales, in which case the continuum limit coincides with the thermodynamic limit of infinite system size. However, as soon as different processes and scales compete, such as hopping, branching, spawning and extinction rates in the present case, the continuum limit is a mere approximation of the original process on the lattice and the choice of (independent) dimensions becomes a claim about which interactions govern the infrared. For example, considering a biased random walk, letting space scale linear in time preserves a drift but removes diffusion, while letting space scale quadratically in time preserves the latter, while the drift velocity diverges.

B. Fourier transform

Throughout the manuscript, we denote the Fourier transform $\mathcal{F}[f(\mathbf{x}, t)]$ of a function $f(\mathbf{x}, t)$ in space $\mathbf{x}$ and time t simply as $f(\mathbf{k}, \omega)$, where the spatial momentum $\mathbf{k}$ is the conjugate of the position $\mathbf{x}$, and the frequency ω is the conjugate of time t. The direct Fourier transform is defined as

$$f(\mathbf{k}, \omega) = \int \mathrm{e}^{\imath\omega t - \imath\mathbf{k}\cdot\mathbf{x}} f(\mathbf{x}, t) \mathrm{d}^d\mathbf{x}\mathrm{d}t, \tag{S10}$$

so that the inverse Fourier transform is

$$f(\mathbf{x}, t) = \int \mathrm{e}^{-\imath\omega t + \imath\mathbf{k}\cdot\mathbf{x}} f(\mathbf{k}, \omega) \text{đ}^d\mathbf{k}\,\text{đ}\omega, \tag{S11}$$

where $\text{đ}^d\mathbf{k} = (1/2\pi)^d \mathrm{d}\mathbf{k}$, $\text{đ}\omega = (1/2\pi)\mathrm{d}\omega$, and d is the spatial dimension.

C. Propagators and couplings

We begin by considering the field-theoretic action $\mathcal{A} = -\int \mathrm{d}^d x \mathrm{d}t\, \mathcal{L}$, where the terms in the Liouvillian $\mathcal{L} = \mathcal{L}_0 + \mathcal{L}_1$ are given by Eqs. (6) and (7), respectively. In order to render the Laplacian term local the action is rewritten in Fourier space, where the momentum $\mathbf{k}$ is the conjugate of position $\mathbf{x}$ and the frequency ω is the conjugate of time t (as defined in Sec. S2 B). The perturbative renormalisation scheme starts by reading off the propagators from the bilinear part, introducing a diagrammatic language as we proceed. For the walkers the bare propagator reads

$$\left\langle \phi(\mathbf{k},\omega)\tilde{\phi}(\mathbf{k}',\omega')\right\rangle_0 = \frac{\mathring{\delta}(\mathbf{k}+\mathbf{k}')\mathring{\delta}(\omega+\omega')}{-\mathring{\imath}\omega + Dk^2 + r} \triangleq \tag{S12}$$

,

where $\mathring{\delta}(\mathbf{k}+\mathbf{k}') = (2\pi)^d \delta(\mathbf{k}+\mathbf{k}')$ denotes a scaled d-dimensional Dirac-δ function, and correspondingly for $\mathring{\delta}(\omega+\omega')$. Diagrammatically, the bare propagator is shown as a straight line. For the tracers the bare propagator becomes

$$\left\langle \psi(\mathbf{k},\omega)\tilde{\psi}(\mathbf{k}',\omega')\right\rangle_0 = \frac{\mathring{\delta}(\mathbf{k}+\mathbf{k}')\mathring{\delta}(\omega+\omega')}{-\mathring{\imath}\omega + \epsilon'} \triangleq \tag{S13}$$

 ,

diagrammatically shown as a wavy line. Both bare propagators carry a positive mass, $r = e - s$ in Eq. (S12) and ϵ' in Eq. (S13), which guarantees causality as the inverse Fourier transform will generate a Heaviside-θ function in time. Both propagators Eqs. (S12) and (S13) do not undergo renormalisation. Finally, the transmutation vertex features in

$$\left\langle \psi(\mathbf{k},\omega)\tilde{\phi}(\mathbf{k}',\omega')\right\rangle_0 = \tau \frac{\mathring{\delta}(\mathbf{k}+\mathbf{k}')\mathring{\delta}(\omega+\omega')}{(-\mathring{\imath}\omega + \epsilon')(-\mathring{\imath}\omega + Dk^2 + r)} \triangleq \tag{S14}$$

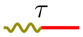

and signals the appearance of a tracer in response to the presence of a walker, as time is to be read from right to left. The non-linear part of the Liouvillian, $\mathcal{L}_1$, contributes with six interaction vertices, which diagrammatically read

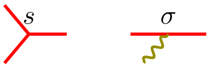

(S15)

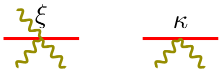

(S16)

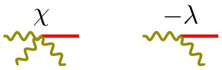

. (S17)

Finally, the observables of the form of Eq. (10) have the diagrammatic structure

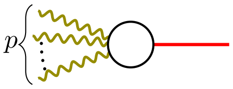

. (S18)

Their scaling in time and finite-size can be extracted from the scaling of the vertex generating function, which is the standard object of field-theoretic renormalisation. In the next section we describe all possible infrared-relevant interactions.

D. Relevant interactions

Whether a particular interaction is allowed by the basic process introduces above is a matter of some topological constraints, which we will discuss in the first part of this section. Whether it is infrared-relevant is determined by its engineering dimension, which we discuss in the second part of this section. Combining topological and engineering constraints will then produce a finite number of interaction vertices to consider. Constraints that avoid certain, otherwise relevant vertices from being generated are preserved under renormalisation.

The general proper vertex

$$\Gamma^{\left[\begin{smallmatrix} m & n \\ p & q \end{smallmatrix}\right]} = \tag{S19}$$

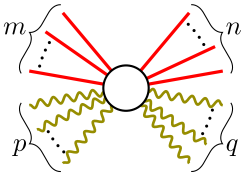

are the one-particle irreducible graphs of the amputated correlation function

$$G^{\left[\begin{smallmatrix} m & n \\ p & q \end{smallmatrix}\right]}(r, D, \tau, s, \sigma, \lambda, \kappa, \chi, \xi; \{\mathbf{k}_1, \dots, \mathbf{k}_{m+n+p+q}; \omega_1, \dots, \omega_{m+n+p+q}\})$$
$$= \Big\langle \underbrace{\phi(\mathbf{k}_1, \omega_1) \dots \phi(\mathbf{k}_m, \omega_m)}_{m \text{ terms}} \underbrace{\psi \dots \psi}_{p \text{ terms}} \underbrace{\tilde{\phi} \dots \tilde{\phi}}_{n \text{ terms}} \underbrace{\tilde{\psi} \dots \tilde{\psi}}_{q \text{ terms}} \Big\rangle \tag{S20}$$

Denoting, where applicable, terms of higher order in non-linear couplings by h.o.t., the bare couplings are the tree-level contributions to the proper vertices:

$$\tau = \Gamma^{\left[\begin{smallmatrix} 0 & 1 \\ 1 & 0 \end{smallmatrix}\right]} + \text{h.o.t.} \qquad s = \Gamma^{\left[\begin{smallmatrix} 2 & 1 \\ 0 & 0 \end{smallmatrix}\right]} \qquad \lambda = \Gamma^{\left[\begin{smallmatrix} 0 & 1 \\ 1 & 1 \end{smallmatrix}\right]} + \text{h.o.t.} \tag{S21a}$$

$$\sigma = \Gamma^{\left[\begin{smallmatrix} 1 & 1 \\ 1 & 0 \end{smallmatrix}\right]} + \text{h.o.t.} \qquad \chi = \Gamma^{\left[\begin{smallmatrix} 0 & 1 \\ 2 & 1 \end{smallmatrix}\right]} + \text{h.o.t.} \qquad \kappa = \Gamma^{\left[\begin{smallmatrix} 1 & 1 \\ 1 & 1 \end{smallmatrix}\right]} + \text{h.o.t.} \qquad \xi = \Gamma^{\left[\begin{smallmatrix} 1 & 1 \\ 2 & 1 \end{smallmatrix}\right]} + \text{h.o.t.} \tag{S21b}$$

Every proper vertex has a number of topological constraints, since any such term needs to arise from the perturbative expansion of the action as a one-particle irreducible (connected, amputated) diagram made from the bare vertices available in the theory. By inspection, we found the following constraints, which we will use to determine all relevant, possible couplings below: Firstly, all non-linear vertices in the field theory (all diagrams except the bare propagator of the tracer particles) have at least one straight leg coming in, $n \geq 1$. Secondly, all vertices have at least as many wavy legs coming out, as come in, $p \geq q$. Thirdly, there are at least as many outgoing legs (wavy or straight), as there are incoming straight legs, $m + p \geq n$.

The engineering dimension of the general proper vertex can be determined from the considerations at the beginning of Section S2 A, using the fact that each proper vertex may be seen as an effective coupling, which, after integration over real time and space, gives rise to a dimensionless contribution to the action, $\mathtt{L}^d\mathtt{T}[\Gamma^{\left[\begin{smallmatrix} m & n \\ p & q \end{smallmatrix}\right]}\tilde{\phi}^m\tilde{\psi}^p\phi^n\psi^q] = 1$, so that

$$[\Gamma^{\left[\begin{smallmatrix} m & n \\ p & q \end{smallmatrix}\right]}] = \mathtt{L}^{d(n+q-1)+2(m-n+2p-2q-1)}\mathtt{A}^{p-q}\mathtt{B}^{m-n+p-q}\mathtt{C}^{n-m-2p+2q+1}. \tag{S22}$$

Demanding that (effective) transmutation τ, branching s and diffusion D may remain relevant at any scale (which amounts to a suitable continuum limit), we set the independent dimensions $\mathtt{A}$, $\mathtt{B}$ and $\mathtt{C}$, respectively, to unity $\mathtt{A} = \mathtt{B} = \mathtt{C} = 1$. The (marginally) infrared-relevant couplings are those whose engineering dimension (in $\mathtt{L}$) is non-positive. At the upper critical dimension $d = d_c = 4$, the inequality $d(n + q - 1) + 2(m - n + 2p - 2q - 1) \leq 0$ gives

$$m + n + 2p \leq 3. \tag{S23}$$

The field theory needs to include all vertices $\Gamma^{\left[\begin{smallmatrix} m & n \\ p & q \end{smallmatrix}\right]}$ with (non-negative) integers m, n, p and q that fulfill Eq. (S23) together with the topological constraints $n \geq 1$, $p \geq q$ and $m + p \geq n$. To find them, we distinguish two cases for Eq. (S23):

- $p = 0 \overset{p \geq q}{\Longrightarrow} q = 0$, then $m + n \leq 3$. Under the topological constraint $m + p \geq n$ there are only two viable solutions: $m = n = 1$, or $m = 2$ and $n = 1$, that correspond to

   and 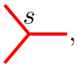

  , (S24)

  the bare propagator for active walkers, and branching of active walkers, respectively.

- $p = 1 \implies m + n \leq 1$. Only the propagator of the immobile particles allows for $n = 0$. Otherwise, $n \geq 1$ requires $m = 0$. The constraint $p \geq q$ leaves only $q = 0$ and $q = 1$. As a result, there are three viable combinations: Firstly, $m = n = 0$ and $q = 1$, secondly, $m = q = 0$ and $n = 1$, thirdly, $m = 0$ and $n = q = 1$, which correspond to

   , 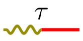

  and 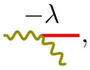

  , (S25)

  the bare propagator of immobile tracer particles, the transmutation vertex and hindrance of spawning, respectively.

Together with the propagators, the vertices in (S24) and (S25) represent all (marginally) relevant couplings at $d = d_c = 4$, consisting of the (bilinear) transmutation, τ, and the interaction vertices s of branching and $-\lambda$ of suppression of spawning.

In the following we perform the renormalisation of the couplings τ and $-\lambda$.

E. Renormalisation of the couplings

As far as the observables in the present work are concerned, the only couplings to consider are τ and λ. Both are renormalised by the same set of loops

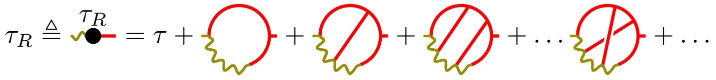

(S26)

and

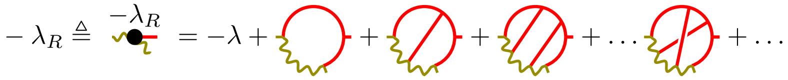

(S27)

where all diagrams are amputated. The subscript R indicates a renormalised quantity, which may still be dimensionfull as in the expression above. Only the non-crossing loop diagrams, such as the first three in Eqs. (S26) and (S27), are easily calculated (see Sec. S3 for details). Of the diagrams in Eqs. (S26) and (S27), the non-crossing ones are summed over by virtue of field-theoretic renormalisation. The last diagram in both Eq. (S26) and Eq. (S27), on the other hand, require further explicit calculation and subsequent summation. The same applies to an infinite number of further crossing diagrams. And yet, because of the Ward-identity (Sec. S2 F)

$$\frac{\partial \tau_R}{\partial \tau} = \frac{\lambda_R}{\lambda} \tag{S28}$$

all exponents can be determined without calculating any of the diagrams explicitly.

As usual in perturbative field theory [S2, S3], the governing non-linearity, here λ, becomes spatially dimensionless by multiplying it by $\mu^{-\epsilon}$, where μ is an arbitrary inverse length scale. In fact, *any* dimensionless coupling involving λ, τ, s, D and μ is proportional to a power of $\lambda s D^{-2}\mu^{-\epsilon}$. Introducing $g = \lambda s \mathcal{U} \mu^{-\epsilon} D^{-2}\Gamma(\epsilon/2)$ with suitable numerical factor $\mathcal{U}$, both couplings λ and τ renormalise identically

$$\tau_R = \tau Z(g) \qquad \text{and} \qquad \lambda_R = \lambda Z(g) \tag{S29}$$

with $Z(g)$ governing the renormalisation of both λ and τ. To one loop and with suitable $\mathcal{U}$, the Z-factor becomes $Z(g) = 1 - g$, see Eqs. (S26) and (S27), and Sec. S3. However, there is no need to determine the precise dependence of Z on g as far as scaling is concerned. It suffices to know that the renormalised, dimensionless

$$g_R = \lambda_R s \mathcal{U} \mu^{-\epsilon} D^{-2}\Gamma(\epsilon/2) \tag{S30}$$

$$= Z\lambda s \mathcal{U} \mu^{-\epsilon} D^{-2}\Gamma(\epsilon/2) \tag{S31}$$

has β-function

$$\beta_g = \frac{\mathrm{d} g_R}{\mathrm{d}\ln\mu} = -\epsilon g_R + g_R \frac{\mathrm{d}\ln Z}{\mathrm{d}\ln\mu} \tag{S32}$$

which implies $\mathrm{d}\ln Z/\mathrm{d}\ln\mu = \epsilon$ at the root $\beta_g(g = g^*) = 0$, irrespective of $\mathcal{U}$ and therefore irrespective of the presence or absence of the crossing diagrams. It follows that $Z \sim \mu^{\epsilon}$ in $d \le 4$ and therefore the effective transmutation rate is $\tau_{\text{eff}} \sim \tau Z \sim \mu^{\epsilon}$. In the limit of $t \to \infty$, for systems of linear size L, the characteristic scale is $\mu \sim L^{-1}$ and thus $\tau_{\text{eff}} \sim L^{-\epsilon}$. With open boundary conditions, the branching walkers visit $\sim L^2$ sites during the course of their lifetimes, leaving behind $\sim \tau_{\text{eff}} L^2 \sim L^{2-\epsilon}$ immobile tracer particles in dimensions greater than 2, so that $\langle a\rangle(t, L) \sim L^{d-2}$. This average is bounded from below by a constant, as at least one site is always visited, so that $\langle a\rangle(t, L)$ approaches a constant below 2 dimensions. As for the time-dependence, the characteristic inverse scale μ is proportional to $t^{-1/2}$ because the dynamical exponent $z = 2$ in $\mu \sim t^{-1/z}$ remains unchanged. It follows that $\langle a\rangle(t, L) \sim t^{(d-2)/2}$.

In the following section, the mean $\langle a\rangle(t, L)$ and higher moments are calculated in greater detail.

F. Ward identity

To identify the Ward-identity, we first state the action

$$\mathcal{A}\left([\phi, \psi, \tilde{\phi}, \tilde{\psi}]; D, r, \epsilon', \tau, s, \lambda\right) = \int \mathrm{d}^d x \mathrm{d}t \left(-\tilde{\phi}\partial_t\phi + D\tilde{\phi}\nabla^2\phi - r\tilde{\phi}\phi - \tilde{\psi}\partial_t\psi - \epsilon'\tilde{\psi}\psi + \tau\tilde{\psi}\phi + s\tilde{\phi}^2\phi - \lambda\tilde{\psi}\psi\phi\right) \tag{S33}$$

after having removed the irrelevant couplings from the Liouvillian $\mathcal{L} = \mathcal{L}_0 + \mathcal{L}_1$, Eqs. (6) and (7). The Ward-identity Eq. (S28) is rooted in a symmetry of the action under shifting $\psi(\mathbf{x}, t)$ by a constant Σ,

$$\mathcal{A}\left([\phi, \psi + \Sigma, \tilde{\phi}, \tilde{\psi}]; D, r, \epsilon', \tau, s, \lambda\right) = \mathcal{A}\left([\phi, \psi, \tilde{\phi}, \tilde{\psi}]; D, r, \epsilon', \tau - \lambda\Sigma, s, \lambda\right) + \Sigma \int \mathrm{d}^d x \mathrm{d}t \, (-\epsilon')\tilde{\psi}(\mathbf{x}, t) \ , \tag{S34}$$

where the last term amounts to a source term, which maintains a density of Σ of immobile particles throughout time and space, as they are subject to continuous decay with (matching) rate ϵ'. To ease notation we write

$$\mathcal{A} = \mathcal{A}\left([\phi, \psi, \tilde{\phi}, \tilde{\psi}]; D, r, \epsilon', \tau, s, \lambda\right) \tag{S35}$$

$$\mathcal{A}' = \mathcal{A}\left([\phi, \psi + \Sigma, \tilde{\phi}, \tilde{\psi}]; D, r, \epsilon', \tau, s, \lambda\right) \tag{S36}$$

$$\mathcal{A}'' = \mathcal{A}\left([\phi, \psi, \tilde{\phi}, \tilde{\psi}]; D, r, \epsilon', \tau - \lambda\Sigma, s, \lambda\right) \tag{S37}$$

so that

$$\mathcal{A}' = \mathcal{A}'' - \epsilon'\Sigma \int \mathrm{d}^d x \mathrm{d}t \, \tilde{\psi}(\mathbf{x}, t) \ , \tag{S38}$$

as well as

$$\langle \bullet \rangle_{\mathcal{A}} = \int \mathcal{D}\Pi \, \bullet \, \mathrm{e}^{\mathcal{A}} \ , \tag{S39}$$

and similarly for the actions $\mathcal{A}'$ and $\mathcal{A}''$. Since ψ is only a dummy variable in this path integral, any expectation over the action $\mathcal{A}$ of an observable involving the field ψ, is identical to the expectation of an observable involving the shifted field $\psi + \Sigma$ over the action $\mathcal{A}'$, for example

$$\left\langle \psi(\mathbf{x}_3, t_3)\psi(\mathbf{x}_2, t_2)\tilde{\psi}(\mathbf{x}_1, t_1)\tilde{\phi}(\mathbf{x}_0, t_0) \right\rangle_{\mathcal{A}} = \left\langle \left(\psi(\mathbf{x}_3, t_3) + \Sigma\right)\left(\psi(\mathbf{x}_2, t_2) + \Sigma\right)\tilde{\psi}(\mathbf{x}_1, t_1)\tilde{\phi}(\mathbf{x}_0, t_0) \right\rangle_{\mathcal{A}'} \ . \tag{S40}$$

To derive the Ward-identity (S28), we consider

$$\begin{aligned} \left\langle \psi(\mathbf{x}, t)\tilde{\phi}(\mathbf{x}_0, t_0) \right\rangle_{\mathcal{A}} = \left\langle \left(\psi(\mathbf{x}, t) + \Sigma\right)\tilde{\phi}(\mathbf{x}_0, t_0) \right\rangle_{\mathcal{A}'} &= \left\langle \psi(\mathbf{x}, t)\tilde{\phi}(\mathbf{x}_0, t_0) \right\rangle_{\mathcal{A}'} + \Sigma \left\langle \tilde{\phi}(\mathbf{x}_0, t_0) \right\rangle_{\mathcal{A}'} \\ &= \left\langle \psi(\mathbf{x}, t)\tilde{\phi}(\mathbf{x}_0, t_0)\mathrm{e}^{-\epsilon' \int \mathrm{d}^d x \mathrm{d}t \, \tilde{\psi}(\mathbf{x}, t)} \right\rangle_{\mathcal{A}''} \end{aligned} \tag{S41}$$

using $\left\langle \tilde{\phi}(\mathbf{x}_0, t_0) \right\rangle_{\mathcal{A}'} = 0$ and Eq. (S38) in the last line. Differentiation with respect to Σ and evaluating at $\Sigma = 0$ then gives

$$0 = -\lambda\partial_\tau \left\langle \psi(\mathbf{x}, t)\tilde{\phi}(\mathbf{x}_0, t_0) \right\rangle_{\mathcal{A}} - \epsilon' \int \mathrm{d}^d x' \mathrm{d}t' \, \left\langle \psi(\mathbf{x}, t)\tilde{\phi}(\mathbf{x}_0, t_0)\tilde{\psi}(\mathbf{x}', t') \right\rangle \tag{S42}$$

as $\mathcal{A}'' = \mathcal{A}$ at $\Sigma = 0$ and the left-hand side of Eq. (S41) is independent of Σ. The integral is most efficiently evaluated after Fourier-transforming, as $\int \mathrm{d}^d x' \mathrm{d}t' \, \tilde{\psi}(\mathbf{x}', t') = \tilde{\psi}(\mathbf{k} = 0, \omega = 0)$ and noting that

$$\left\langle \psi(\mathbf{k}', \omega')\tilde{\psi}(\mathbf{k} = 0, \omega = 0) \right\rangle = \frac{1}{\epsilon'}\delta\!\!\!{}^-(\omega')\delta\!\!\!{}^-(\mathbf{k}') \tag{S43}$$

whenever $\tilde{\psi}(\mathbf{k} = 0, \omega = 0)$ is paired up with any internal field $\psi(\mathbf{k}', \omega')$. Dividing out two bare propagators, the right-hand side of Eq. (S42) consists of the amputated diagrams shown in Eq. (S26) and Eq. (S27), so that

$$0 = -\lambda\partial_\tau \tau_R + \frac{\epsilon'}{\epsilon'}\lambda_R \ , \tag{S44}$$

the desired identity Eq. (S28).

G. Calculating scaling of higher-order correlation functions

The scaling of higher-order correlation functions is derived, within the field theory, from the solution of the Callan–Symanzik equation [S2] for the general proper vertex Eq. (S19), from which the scaling of the moments of the total number of distinct sites visited follow, Eq. (2). From dimensional analysis (Sec. S2 A), and by introducing a bare scale μ_0, related to μ by $\mu = \mu_0 \ell$, the general proper vertex, Eq. (S19), then satisfies

$$\Gamma^{\left[\begin{smallmatrix} m & n \\ p & q \end{smallmatrix}\right]}(r, D, \tau, s, \sigma, \lambda, \kappa, \chi, \xi; \{\mathbf{k}; \omega\}) \\ = \ell^{-d(n+q-1)-2(m-n+2p-2q-1)+(p-q)\gamma_\tau} \Gamma^{\left[\begin{smallmatrix} m & n \\ p & q \end{smallmatrix}\right]} \left(\frac{r}{\ell^2}, D, \tau, s, \sigma, \lambda, \kappa, \chi, \xi; \left\{ \frac{\mathbf{k}}{\ell}; \frac{\omega}{\ell^2} \right\} \right), \tag{S45}$$

asymptotically in small ℓ and provided that r is close enough to the critical point, $r_c = 0$. For the transmutation vertex, where $p = n = 1$ and $q = m = 0$, we find

$$\Gamma^{\left[\begin{smallmatrix} 0 & 1 \\ 1 & 0 \end{smallmatrix}\right]}(r, D, \tau, s, \sigma, \lambda, \kappa, \chi, \xi; \{\mathbf{k}; \omega\}) = \ell^{\gamma_\tau} \Gamma^{\left[\begin{smallmatrix} 0 & 1 \\ 1 & 0 \end{smallmatrix}\right]} \left(\frac{r}{\ell^2}, D, \tau, s, \sigma, \lambda, \kappa, \chi, \xi; \left\{ \frac{\mathbf{k}}{\ell}; \frac{\omega}{\ell^2} \right\} \right), \tag{S46}$$

with $\gamma_\tau = \varepsilon = 4 - d$. Generally, for observables of the form Eq. (S18), where $n = 1$ and $q = m = 0$ we have

$$\Gamma^{\left[\begin{smallmatrix} 0 & 1 \\ p & 0 \end{smallmatrix}\right]}(r, D, \tau, s, \sigma, \lambda, \kappa, \chi, \xi; \{\mathbf{k}; \omega\}) = \ell^{4(1-p)+p\gamma_\tau} \Gamma^{\left[\begin{smallmatrix} 0 & 1 \\ p & 0 \end{smallmatrix}\right]} \left(\frac{r}{\ell^2}, D, \tau, s, \sigma, \lambda, \kappa, \chi, \xi; \left\{ \frac{\mathbf{k}}{\ell}; \frac{\omega}{\ell^2} \right\} \right). \tag{S47}$$

The scaling of the first moment of the number of distinct sites visited, $\langle a(t) \rangle$, as function of time, t, can be obtained by analysing the scaling of

$$\langle a(t) \rangle = \int \mathrm{d}^d\mathbf{x} \left\langle \psi(\mathbf{x}, t) \tilde{\phi}(\mathbf{x}_0, 0) \right\rangle \tag{S48}$$

$$\triangleq \int đ\omega đ\omega_0 \mathrm{e}^{-\mathring{\imath}\omega t} \Big|_{\mathbf{k}=0} \tag{S49}$$

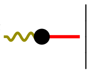

$$= \int đ\omega \mathrm{e}^{-\mathring{\imath}\omega t} \frac{1}{-\mathring{\imath}\omega + \epsilon'} \Gamma^{\left[\begin{smallmatrix} 0 & 1 \\ 1 & 0 \end{smallmatrix}\right]} \frac{1}{-\mathring{\imath}\omega + r}. \tag{S50}$$

According to Eq. (S46), $\Gamma^{\left[\begin{smallmatrix} 0 & 1 \\ 1 & 0 \end{smallmatrix}\right]}$ scales like

$$\Gamma^{\left[\begin{smallmatrix} 0 & 1 \\ 1 & 0 \end{smallmatrix}\right]}(L^{-2}, D, \tau, s, \sigma, \lambda, \kappa, \chi, \xi; \{\mathbf{k}; \omega\}) = L^{-\gamma_\tau} \Gamma^{\left[\begin{smallmatrix} 0 & 1 \\ 1 & 0 \end{smallmatrix}\right]} \left(1, D, \tau, s, \sigma, \lambda, \kappa, \chi, \xi; \left\{ \mathbf{k}L; \omega L^2 \right\} \right), \tag{S51}$$

if we identify $r \sim L^{-2}$ and $\ell \sim L^{-1}$, which means that the effective transmutation rate scales like $L^{-\varepsilon}$ in large linear system size L, as $\gamma_\tau = \varepsilon = 4 - d$. In long time t, the integral over ω in Eq. (S50) has the effect of evaluating $\Gamma^{\left[\begin{smallmatrix} 0 & 1 \\ 1 & 0 \end{smallmatrix}\right]} \frac{1}{\mathring{\imath}\omega + r}$ at $\omega = 0$, because

$$\lim_{t\to\infty} \lim_{\epsilon'\to 0} \int_{-\infty}^{\infty} \mathrm{e}^{-\mathring{\imath}\omega t} \frac{1}{-\mathring{\imath}\omega + \epsilon'} f(\omega) = f(0) \tag{S52}$$

provided $f(\omega)$ has no pole at 0.

It follows that

$$\lim_{t\to\infty} \langle a(t) \rangle \propto L^{2-\epsilon}. \tag{S53}$$

For higher moments, on the basis of Eq. (S47) we find

$$\lim_{t\to\infty} \langle a^p(t) \rangle \propto L^2 L^{pd-4} \Gamma^{\left[\begin{smallmatrix} 0 & 1 \\ p & 0 \end{smallmatrix}\right]} (1, D, \tau, s, \sigma, \lambda, \kappa, \chi, \xi; \{0, 0\}). \tag{S54}$$

We thus recover the finite-size scaling results Eqs. (2b) and (3b) of Section II for the p-th moment of the volume explored by a branching random walk

$$\lim_{t\to\infty} \langle a^p(t) \rangle \propto \begin{cases} L^{dp-2} & \text{if } \varepsilon > 0 \\ L^{4p-2} & \text{if } \varepsilon < 0 \end{cases} \tag{S55}$$

where $\varepsilon > 0$ and $\varepsilon < 0$ separate regions below and above the upper critical dimension, $d_c = 4$, respectively. The dimensionality of the embedding space enters only below the upper critical dimension. Above the upper critical dimension, fluctuations and interactions become asymptotically irrelevant and the process can be considered as free.

The above analysis is easily extended to scaling in time, using $t \propto \mu^{-z}$ with $z = 2$ as the relevant scale, thereby reproducing Eqs. (2a) and (3a).

S3. LOOP INTEGRALS

The non-crossing diagrams, such as the first three in Eqs. (S26) and (S27), are calculated through the integral

$I_\tau =$ 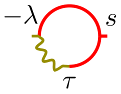

$$= \int \text{đ}^d k \text{đ}\omega \, \frac{\tau}{-\mathring{\imath}\omega + \epsilon'} \frac{1}{\omega^2 + (Dk^2 + r)^2} = \tau \frac{1}{2} \frac{r^{-\varepsilon/2}}{(4\pi D)^{d/2}} \Gamma(\varepsilon/2) \; , \tag{S56}$$

and (essentially identical)

$I_{-\lambda} =$ 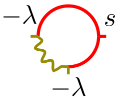

$$= \int \text{đ}^d k \text{đ}\omega \, \frac{-\lambda}{-\mathring{\imath}\omega + \epsilon'} \frac{1}{\omega^2 + (Dk^2 + r)^2} = -\lambda \frac{1}{2} \frac{r^{-\varepsilon/2}}{(4\pi D)^{d/2}} \Gamma(\varepsilon/2) \; , \tag{S57}$$

where the lower part of the loop carries the coupling τ in case of contributing to τ or the coupling $-\lambda$ and an incoming wavy leg in case of contributing to λ. The integration measure is $\text{đ}^d k \text{đ}\omega = \mathrm{d}^d k \mathrm{d}\omega / (2\pi)^{d+1}$.

S4. GENERALISATION TO k OFFSPRING

In this section we extend the field-theoretic results presented above to the case where the offspring number is a random number and show that it lies in the same universality class as binary branching [S4, S5]. Instead of two distinct processes for branching into two active walkers (with rate s above) and getting extinguished (with rate e above) we consider the latter as branching into $k = 0$ walkers and generalise the former to branching into any number k of walkers. Each of these processes may occur with rate σ_k, which can always be written as $\sigma_k = \sigma p_k$ with p_k the normalised probability for branching into k walkers and σ the rate with which any such processes take place.

The two contributions $\mathcal{P}_s$, Eq. (S2), and $\mathcal{P}_e$, Eq. (S3), are thus subsumed and generalised by

$$\dot{\mathcal{P}}_c(\{n\}, \{m\}; t) = \sigma \sum_{k=0}^{\infty} \sum_{\mathbf{x}} p_k \Big((n_\mathbf{x} - k + 1) \mathcal{P}(\{\ldots, n_\mathbf{x} - k + 1, \ldots\}, \{m\}; t) - n_\mathbf{x} \mathcal{P}(\{n\}, \{m\}; t) \Big), \tag{S58}$$

which allows for p_1, but the process of branching into a single particle has no bearing on the master equation.

In the field theory, the mass of the bare propagator for active walkers becomes [S6]

$$r = -\sigma \sum_{k=0}^{\infty} p_k (k - 1) = \sigma (1 - \bar{k}), \tag{S59}$$

where $\bar{k} = \sum_{k=0}^{\infty} p_k k$ is the average offspring number, which again, defines a subcritical ($r > 0$), a critical ($r = 0$), and a supercritical ($r < 0$) regime.

In the case of generalised branching, the non-linear part of the action contains contributions of the form $\tilde{\phi}^k \phi$ for all $k \geq 2$ as soon as there is any $k \geq 2$ with $p_k > 0$ [S6]. Terms with $k > 2$, however, turn out to be infrared irrelevant, as their couplings have dimension $\mathtt{B}^{k-1} \mathtt{C}^{2-k} \mathtt{L}^{2(k-2)}$. The field theoretic results above for binary branching therefore govern also branching processes with generalised offspring distribution.

S5. EXTENSION TO GENERAL GRAPHS

In this section we provide further details about the extension of our results to general graphs. The loops integrated over in Eqs. (S56) and (S57) are in fact integrals over the spectrum of the Laplacian accounting for the diffusion on the graph considered. Generalising to arbitrary graphs, the Laplacian is to be replaced by a lattice-Laplacian and the integral in Eqs. (S56) and (S57) by a suitable sum or, equivalently, an integral with suitable spectral density. In fact, the d-dimensional integral in Eqs. (S56) and (S57) can be seen as an integral over all distinct eigenvalues $\mathbf{k}^2$ of the Laplacian entering with weight $w(k)\mathrm{d}k = S_d k^{d-1} \mathrm{d}k$ with $S_d = 2\pi^{d/2}/\Gamma(d/2)$. On regular lattices, their Hausdorff dimension d coincides with the spectral dimension d_s characteristing, in particular, the small k asymptote of $w(k) \sim k^{d_s - 1}$. Replacing $\int \mathrm{d}^d k$ by $\int \mathrm{d}k \, w(k)$ suggests that the results derived above remain valid by replacing d by d_s, in order to recover the scaling of the various observables in arbitrary graphs with spectral dimension d_s.

The replacement $d \to d_s$ hinges crucially on the fact that d_s characterises the scaling of the spectral density of the Laplacian. If this operator itself renormalises, then a different spectral density may be needed. In other words, d_s may not be the correct dimension if the Laplacian renormalises, *i.e.* if the anomalous dimension does not vanish, $\eta \neq 0$ [S7]. This argument relies on the assumption that vertices such as Eq. (S19) preserve momentum, that is integrals of the form

$$I_n(\mathbf{k}_1, \mathbf{k}_2, \dots, \mathbf{k}_n) = \int \mathrm{d}^d x \, u_{\mathbf{k}_1}(x) u_{\mathbf{k}_2}(x) \dots u_{\mathbf{k}_n}(x) \tag{S60}$$

over eigenfunctions $u_{\mathbf{k}}(x)$ of the Laplacian with eigenvalue $\mathbf{k} \cdot \mathbf{k}$ vanish for off-diagonal terms, i.e. whenever $\mathbf{k}_1 + \mathbf{k}_2 + \dots + \mathbf{k}_n \neq 0$. This condition can be further relaxed by demanding merely that off-diagonal terms are sub-leading as observed in the presence of boundaries [S1, S8].

Considering only graphs which are translationally invariant such that the indices $\mathbf{j}_m$ of the q neighbours $m = 1, \dots, q$ of any node $\mathbf{i}$ can be determined by adding the same set of translational lattice vectors, $\mathbf{d}_1, \dots, \mathbf{d}_q$, such that $\mathbf{j}_m = \mathbf{i} + \mathbf{d}_m$, it is easy to show that the Laplacian has exponential eigenfunctions and any of their products are an eigenfunction as well, so that $I_n(\mathbf{k}_1, \mathbf{k}_2, \dots, \mathbf{k}_n) = I_2(\mathbf{k}_1, \mathbf{k}_2 + \dots + \mathbf{k}_n)$, which vanishes by orthogonality for any $\mathbf{k}_1 + \mathbf{k}_2 + \dots + \mathbf{k}_n \neq \mathbf{0}$, i.e. the assumption of momentum conservation mentioned above is fulfilled.

S6. NUMERICS FOR THE SCALING OF MOMENTS

The scaling of the moments $\langle a^p \rangle (t, L)$ for $p = 1, 2, 3, \dots, 5$, as function of time t in the limit $L \to \infty$, and as function of the system size L in the limit $t \to \infty$ were obtained from numerical Monte Carlo simulations and fitted against a power-law

$$f(x) = Ax^B \tag{S61}$$

and a power-law with corrections of the form

$$g(x) = Ax^B + Cx^{B-1/2}. \tag{S62}$$

The fitting parameter B in Eqs. (S61) and (S62) provides the estimates of the exponents that characterise the scaling of the moments in time t and sistem size L (or N, see main text), by fitting the numerical estimates against $f(x)$ and $g(x)$, with x replaced by t and L, respectively. At large times the moments display plateauing due to finite-size effects.

For the scaling in system size L, we fitted the data for the latest time point available against Eq. (S61) and used the estimates of A and B as the initial values for a fit against Eq. (S62), which gave the final estimates of the finite-size scaling exponents.

For the scaling in time t, we fitted data for the largest system, of size $L = L_{\max}$. The fitting range in t for each moment was determined systematically as follows. To remove the time-point affected by the finite-size effects, we defined the upper bound of the fitting range as the time t^{up} for which the lowest moment displaying algebraic divergence ($p = p_{\mathrm{low}}$) reached a value of half the maximum value in the plateau, *i.e.* $\langle a^{p_{\mathrm{low}}} \rangle (t^{\mathrm{up}}, L_{\max}) = \max_t \left(\langle a^{p_{\mathrm{low}}} \rangle (t, L_{\max}) \right) / 2$. For the preferential attachment network the plateau was observed to occur at an earlier time point than t^{up}, probably due to the high connectivity of the networks, so we set the upper bound to $t^{\mathrm{up}}_{pa} = (1/5) \max(\langle a^k \rangle)$, in this case.

To find the lower bound t_{low} of the fitting range in t we fitted both equations, (S61) and (S62), to the data for $L_{\max}$. We define $\hat{f}_{[t^*, t^{\mathrm{up}}]}(t)$ and $\sigma^{\hat{f}}_{[t^*, t^{\mathrm{up}}]}(t)$ as the values and errors, respectively, of fitting Eq. (S61) to the data in the range $t \in [t^*, t^{\mathrm{up}}]$, and $\hat{g}_{[t^*, t^{\mathrm{up}}]}(t)$ and $\sigma^{\hat{g}}_{[t^*, t^{\mathrm{up}}]}(t)$ as the values and errors, respectively, of fitting Eq. (S62) to the same data set and range. Further, we define $N_{[t^*, t^{\mathrm{up}}]}$ as the number of data points within the fitting interval $[t^*, t^{\mathrm{up}}]$. The lower bound for the time range t_{low} is the earliest time at which both fitting models (S61) and (S62) agree within errors, that is

$$t_{\mathrm{low}} = \min \left\{ t^* : |\hat{f}_{[t^*, t^{\mathrm{up}}]}(t^*) - \hat{g}_{[t^*, t^{\mathrm{up}}]}(t^*)| \leq \sqrt{N_{[t^*, t^{\mathrm{up}}]}} \max \left(\sigma^f_{[t^*, t^{\mathrm{up}}]}(t^*), \sigma^g_{[t^*, t^{\mathrm{up}}]}(t^*) \right) \right\}. \tag{S63}$$

Where we account for correlations between estimates of moments by rescaling the error by the square root of the number of data points in the fitting range, $N_{[t^*, t^{\mathrm{up}}]}$. The exponents characterising the time depenence of the moments are determined by fitting the data in the range $[t^*, t^{\mathrm{up}}]$ against Eq. (S62).

The fitting of the power laws, Eqs. (S61) and (S62), was done by means of the Levenberg-Marquardt algorithm [S9]. In table S1 and S2 we report the numerical results for the asymptotic scaling in time, $\langle a^p \rangle (t) \sim t^{\alpha_p}$, and in system size, $\langle a^p \rangle (t) \sim L^{\beta_p}$, provided these observables display an algebraic divergence.

Scaling of visited sites in time

exponent	d=1		d=2		d=3		d=5		S.C.		R.T.		P.A.	
	num	theo	num	theo	num	theo	num	theo	num	theo	num	theo	num	theo
α_1					0.47(2)	1/2	1.0(2)	1					1.0(1)	1
α_2			0.98(3)	1	2.0(1)	2	2.9(3)	3	0.81(5)	0.86	0.35(7)	1/3	2.8(2)	3
α_3	0.48(4)	1/2	2.0(1)	2	3.5(1)	7/2	4.8(4)	5	1.71(7)	1.79	0.9(1)	1	4.8(4)	5
α_4	1.0(1)	1	2.9(1)	3	5.0(2)	5	6.7(7)	7	2.62(10)	2.72	1.6(2)	5/3	6.6(5)	7
α_5	1.5(1)	3/2	3.9(1)	4	6.4(2)	13/2	9(1)	9	3.54(14)	3.66	2.2(4)	7/3	8.5(9)	9
mean gap	0.5(1)	1/2	1.0(1)	1	1.5(2)	3/2	2.0(5)	2	0.91(9)	0.93	0.6(2)	2/3	1.9(4)	2

Table S1: Scaling in time, $\langle a^p \rangle (t) \sim t^{\alpha_p}$, of the p-th moment of the number of distinct sites visited for regular lattices of integer dimension, d, as indicated, and for the Sierpinski carpet (S.C., $d_s \approx 1.86$), the random tree (R.T., $d_s = 4/3$), and preferential attachment (P.A., $d_s > 4$) networks. The columns marked *num* shows the numerical results, the columns marked *theo* show theoretical results according to Eqs. (2a) with d replaced by the spectral dimension d_s where applicable. The row marked *mean gap* show the average gap-exponent, $(1/p)\sum_{i=1}^{p}(\alpha_{i+1} - \alpha_i)$, for the corresponding lattice.

Scaling of visited sites by a BRW as function of the system size

exponent	d=1		d=2		d=3		d=5		S.C.		R.T.		P.A.	
	num	theo	num	theo	num	theo	num	theo	num	theo	num	theo	num	theo
β_1					0.97(4)	1	1.9(2)*	2					0.49(1)	1/2
β_2			2.1(2)	2	3.9(1)*	4	5.7(5)*	6	1.9(1)	1.72	0.58(6)	1/2	1.49(1)	3/2
β_3	0.96(4)	1	4.2(3)	4	6.8(2)*	7	10(1)	10	3.8(2)	3.59	1.6(1)	3/2	2.49(2)	5/2
β_4	1.93(7)	2	6.1(3)	6	9.8(3)*	10	14(2)	14	5.7(3)	5.45	2.6(2)	5/2	3.49(2)	7/2
β_5	2.93(8)	3	8.1(4)	8	12.7(4)*	13	17(3)	18	7.5(4)	7.31	3.7(2)	7/2	4.49(2)	9/2
mean gap	0.9(1)	1	2.0(3)	2	2.9(2)	3	4(1)	4	1.9(3)	1.86	1.0(1)	1	1.00(2)	1
fit range	[255, 4095]		[15, 127]		[7, 127]		[7, 31]		[9, 243]		$[2^6-1, 2^{12}-1]$		$[2^{14}-1, 2^{19}-1]$	

Table S2: Scaling, $\langle a^p \rangle (t) \sim L^{\beta_p}$, of the p-th moment of the number of distinct sites visited as function of the system size L, for regular lattices of integer dimension d as indicated and for the Sierpinski carpet (S.C., $d_s \approx 1.86$). The columns marked *num* show the numerical results, the columns marked *theo* show theoretical results according to Eq. (2b), for regular lattices, and S.C. (with d replaced by the spectral dimension d_s), and according to Eq. (5) for random tree (R.T.) and preferential attachment (P.A.) The row marked *mean gap* shows the average gap-exponent, $(1/p)\sum_{i=1}^{p}(\beta_{i+1} - \beta_i)$, for the corresponding lattice. *Goodness of fit < 0.05.

SUPPLEMENTARY FIGURE

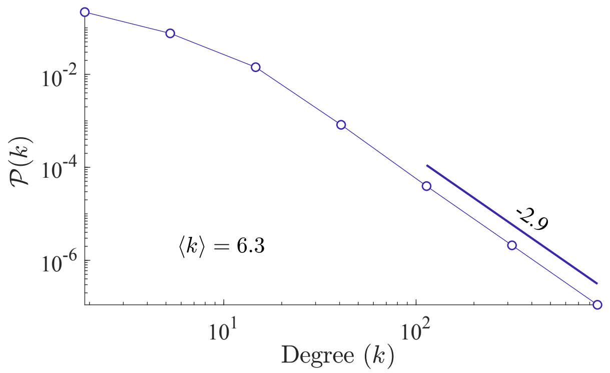

Figure S1: **Degree distribution of the preferential attachment networks** used for the simulations presented in Sec. III

[S1] Nekovar, S. & Pruessner, G. A field-theoretic approach to the wiener sausage. *J. Stat. Phys.* **163**, 604–641 (2016).

[S2] Täuber, U. C. *Critical Dynamics A Field Theory Approach to Equilibrium and Non-Equilibrium Scaling Behavior* (Cambridge University Press, Cambridge, England, 2014).

[S3] Le Bellac, M. *Quantum and Statistical Field Theory [Phenomenes critiques aux champs de jauge, English]* (Oxford University Press, New York, NY, USA, 1991). Translated by G. Barton.

[S4] Aldous, D. The continuum random tree iii. *Ann. Probab.* **21**, 248–289 (1993).

[S5] Le Gall, J.-F. Random trees and applications. *Probab. Surv.* **2**, 245–311 (2005).

[S6] Garcia-Millan, R., Pausch, J., Walter, B. & Pruessner, G. Field-theoretic approach to the universality of branching processes. *Phys. Rev. E* **98**, 062107 (2018).

[S7] Burioni, R. & Cassi, D. Random walks on graphs: ideas, techniques and results. *J. Phys. A: Math. Gen.* **38**, R45–R78 (2005).

[S8] Diehl, H. W. & Schmidt, F. M. The critical Casimir effect in films for generic non-symmetry-breaking boundary conditions. *New J. Phys.* **13**, 123025 (2011).

[S9] Press, W. H., Teukolsky, S. A., Vetterling, W. T. & Flannery, B. P. *Numerical Recipes in C* (Cambridge University Press, New York, NY, USA, 1992), 2nd edn.
